# Effect Of Dual sEH/COX-2 Inhibition on Allergen-Induced Airway Inflammation

**DOI:** 10.3389/fphar.2019.01118

**Published:** 2019-09-27

**Authors:** Mythili Dileepan, Stephanie Rastle-Simpson, Yana Greenberg, Dayanjan S. Wijesinghe, Naren Gajenthra Kumar, Jun Yang, Sung Hee Hwang, Bruce D. Hammock, P. Sriramarao, Savita P. Rao

**Affiliations:** ^1^Department of Veterinary & Biomedical Sciences, University of Minnesota, St. Paul, MN, United States; ^2^Department of Pharmacotherapy and Outcomes Sciences, School of Pharmacy, Virginia Commonwealth University, Richmond, VA, United States; ^3^Department of Entomology, Nematology and Comprehensive Cancer Center, University of California, Davis, CA, United States

**Keywords:** eosinophilia, allergic airway inflammation, pharmacological inhibition, COX-2, soluble epoxide hydrolase

## Abstract

Arachidonic acid metabolites resulting from the cyclooxygenase (COX), lipoxygenase, and cytochrome P450 oxidase enzymatic pathways play pro- and anti-inflammatory roles in allergic airway inflammation (AAI) and asthma. Expression of COX-2 and soluble epoxide hydrolase (sEH) are elevated in allergic airways and their enzymatic products (e.g., prostaglandins and diols of epoxyeicosatrienoic acids, respectively) have been shown to participate in the pathogenesis of AAI. Here, we evaluated the outcome of inhibiting the COX-2 and sEH enzymatic pathways with a novel dual inhibitor, PTUPB, in *A. alternata*-induced AAI. Allergen-challenged mice were administered with 10 or 30 mg/kg of PTUPB, celecoxib (selective COX-2 inhibitor), *t*-TUCB (selective sEH inhibitor) or vehicle daily by gavage and evaluated for various features of AAI. PTUPB and *t*-TUCB at 30 mg/kg, but not celecoxib, inhibited eosinophilic infiltration and significantly increased levels of anti-inflammatory EETs in the lung tissue of allergen-challenged mice. *t*-TUCB significantly inhibited allergen-induced IL-4 and IL-13, while a less pronounced reduction was noted with PTUPB and celecoxib. Additionally, *t*-TUCB markedly inhibited eotaxin-2, an eosinophil-specific chemokine, which was only marginally reduced by PTUPB and remained elevated in celecoxib-treated mice. PTUPB or *t*-TUCB administration reversed allergen-induced reduction in levels of various lipid mediators in the lungs, with only a minimal effect noted with celecoxib. Despite the anti-inflammatory effects, PTUPB or *t*-TUCB did not reduce allergen-induced airway hyperresponsiveness (AHR). However, development of structural changes in the allergic airways, such as mucus hypersecretion and smooth muscle hypertrophy, was significantly inhibited by both inhibitors. Celecoxib, on the other hand, inhibited only airway smooth muscle hypertrophy, but not mucus hypersecretion. In conclusion, dual inhibition of COX-2 and sEH offers no additional advantage relative to sEH inhibition alone in attenuating various features associated with *A. alternata*-induced AAI, while COX-2 inhibition exerts only moderate or no effect on several of these features. Dual sEH/COX-2 inhibition may be useful in treating conditions where eosinophilic inflammation co-exists with pain-associated inflammation.

## Introduction

Allergic airway inflammation (AAI), including allergic asthma, is associated with increased pulmonary recruitment of inflammatory cells, especially eosinophils, along with airway hyperresponsiveness (AHR) and elevated levels of Th2 cytokines, pro-inflammatory chemokines and growth factors (Hamid and Tulic, 2009; Lambrecht and Hammad, 2015). Arachidonic acid (AA) metabolites such as prostaglandins (PGs), leukotrienes (LTs) and epoxyeicosatrienoic acids (EETs) resulting from the cyclooxygenase (COX), lipoxygenase (LOX) and cytochrome P450 oxidase (CYP) enzymatic pathways, respectively (Hanna and Hafez, 2018), are known to participate in various aspects of AAI and asthma (Norton et al., 2012; Claar et al., 2015; Yang et al., 2015; Schauberger et al., 2016; Debeuf and Lambrecht, 2018). COX-2 expression is increased in the airways of asthmatic subjects (Sousa et al., 1997; Profita et al., 2003) and in the lungs of allergen-challenged mice (Shiraishi et al., 2008; Herrerias et al., 2009). Studies to understand the importance of COX-2 during AAI in animal models using COX-2 knock-out (KO) mice or a COX-2 inhibitor have not been straightforward, with differing outcomes noted among various studies (reviewed in Peebles, 2018). Products of the COX pathway such as PGD2 and PGE2 are elevated during AAI and known to participate in the pathogenesis of airway allergic disease in humans (Profita et al., 2003; Fajt et al., 2013; Peebles, 2018) and animal models (Shiraishi et al., 2008; Herrerias et al., 2009). Additionally, COX-2-derived PGs enhance Th17 cell differentiation of naive CD4^+^ T cells during allergic inflammation in mice (Li et al., 2011) and PGD2 increases production of the Th2 cytokine IL-13 from human peripheral blood ILC2s in the presence of IL-33 and IL-25, suggesting a role for lipid mediators in regulating ILC2 function (Barnig et al., 2013; Doherty and Broide, 2018).

EETs are a class of lipid mediators generated by the CYP P450 oxidase pathway of AA metabolism that function as anti-inflammatory mediators (Christmas, 2015). However, they are rapidly converted to diol derivatives, i.e., dihydroxyeicosatrienoic acids (DiHETs) by the enzyme soluble epoxide hydrolase (sEH); thus, sEH has a pro-inflammatory role in various inflammation models (Morisseau and Hammock, 2013; Zhang et al., 2014). Expression of sEH is increased in bronchial biopsies from asthmatic patients relative to normal subjects (Morin et al., 2010). Additionally, studies have shown that inhibition of sEH with a selective inhibitor attenuates ovalbumin (OVA)-induced eosinophilia, reduces levels of Th2 cytokines and chemokines, and improves airway resistance and compliance in mice (Yang et al., 2015). Along these lines, our recent studies have shown that expression of sEH is induced by food allergens in the gastrointestinal (GI) tract and that inhibition of sEH attenuates allergen-induced eosinophilia, mast cell recruitment and mucus secretion in the jejunum (Bastan et al., 2018). As such, identification of inhibitors targeting the COX-2 and sEH enzymatic pathways in the context of allergic airway disease is likely to yield valuable information contributing to the development of novel strategies to manage this disease.

Recent studies have shown that inhibition of the COX-2 and sEH pathways using the dual inhibitor PTUPB (4-(5-phenyl-3-{3-[3-(4-trifluoromethyl-phenyl)-ureido]-propyl}-pyrazol-1-yl)-benzenesulfonamide) has therapeutic value in reducing inflammation in models of cancer (Zhang et al., 2014; Li et al., 2017; Gartung et al., 2019) and diabetes (Hye Khan et al., 2016). Here, we investigated the effect of dual inhibition of the sEH and the COX-2 pathways in reducing eosinophilia and airway inflammation in an experimental model of AAI induced by *Alternaria alternata*.

## Materials and Methods

### Mouse Model of Allergy

C57BL/6 mice (8–10 weeks, male and female) were challenged with 50 µg of an extract of *A. alternata* (Greer Laboratories, Lenoir, NC) in 50 µl of PBS on days 0, 3 and 6 or with PBS alone (control) as described previously (Ha et al., 2013). *A. alternata*-challenged mice were administered daily with PTUPB, a sEH/COX-2 dual inhibitor, *t*-TUCB (*trans*-4-{4-[3-(4-trifluoromethoxyphenyl)-ureido]-cyclohexyloxy}-benzoic acid), a highly selective sEH inhibitor, or celecoxib, a selective COX-2 inhibitor (Sigma-Aldrich Corp., St. Louis, MO). Inhibitors were dissolved in 80% Kollisolv® PEG E 300 (Sigma-Aldrich Corp., prepared in sterile water). 80% Kollisolv® PEG E 300 alone was used as the vehicle control. Synthesis and structure-activity relationship of PTUPB and *t*-TUCB have been previously described (Hwang et al., 2007; Hwang et al., 2011). Inhibitors at a dosage of 10 or 30 mg/kg were administered by gavage one hour before airway allergen challenge and mice were sacrificed 24 hours after the last challenge. Control (PBS) mice were administered with 80% Kollisolv® PEG E 300. All animal studies were performed following standards and procedures approved by the Institutional Animal Care and Use Committee at the University of Minnesota.

### Sample Collection and Analysis

At the end of the study, lungs of mice were lavaged with 1 ml saline. Total and differential cell counts in the bronchoalveolar lavage fluid (BALF) were determined based on morphologic and histologic criteria after staining cytocentrifuged samples with Hema 3 System (Thermo Fisher Scientific, Pittsburgh, PA). BALF supernatants were stored at −80°C for later analysis. Right lungs were snap-frozen and left lungs were perfused with 4% paraformaldehyde (PFA) to preserve pulmonary structure, fixed in 4% PFA and paraffin embedded. Bone marrow (BM) was collected and used to determine eosinophil counts based on cell morphology after Hema 3 staining.

### Histology

Lung tissue sections (4 µm thick) were stained with Hematoxylin and Eosin (Leica Biosystems, Inc., Buffalo Grove, IL) to determine cellular infiltration. Analysis of lung tissue for infiltrated eosinophils was performed by immunohistochemical staining of sections for eosinophil-specific major basic protein (MBP) with rat mAb against murine MBP as described in our previous studies (Zuberi et al., 2009). Positively stained cells (reddish brown) were counted (at 400× magnification) in randomly selected non-overlapping fields (approximately 20 fields/mouse) and expressed as the average number of MBP-positive cells/microscopic field. Sections were stained with Periodic acid-Schiff’s (PAS) reagent (Sigma-Aldrich) to detect airway mucus production. PAS-positive areas (dark pink) in horizontally sectioned airways were quantitated using ImageJ image analysis program (Abramoff et al., 2004) and expressed as μm PAS-positive area/100 μm basement membrane length (BML) (Bahaie et al., 2012). Expression of α-smooth muscle actin (α-SMA) was evaluated by immunohistochemistry using mAb against α-SMA (2 μg/ml, Sigma-Aldrich) and area of the positively stained peribronchial smooth muscle layer was quantitated from captured images using ImageJ as described (Ge et al., 2010). For immunohistochemical detection of COX-2, lung sections were first permeabilized using 0.4% Triton X-100 in Tris-buffered saline containing 0.1% Tween 20 (TBST) and 1% horse serum before blocking in 5% horse serum and then incubating with a polyclonal antibody against COX-2 [5.5 μg/ml (Novus Biologicals, Centennial, CO), 3 h at room temperature]. VECTASTAIN Elite ABC kit containing biotinylated anti-rabbit IgG (Vector Laboratories, Burlingame, CA), and the Peroxidase AEC (3-amino-9-ethylcarbazole) substrate kit (Vector Laboratories) were used for detection of bound antibodies. Expression of sEH in lungs of OVA-challenged mice (Ge et al., 2016) was detected as described previously (Bastan et al., 2018). In all cases, stained slides were examined using a Nikon Microphot EPI-FL microscope and images were captured with an Olympus DP71 camera.

### Measurement of Airway Responsiveness

Pulmonary function was assessed in anesthetized control and *A. alternata*-challenged mice with or without inhibitor treatment using the FinePointe^TM^ RC system (Buxco, Wilmington, NC) as described in our previous studies (Bahaie et al., 2012). Changes in pulmonary resistance (R_L_) were monitored continuously in response to saline followed by increasing concentrations of inhaled methacholine (3–50 mg/ml) nebulized for 18–20 s and expressed as mean R_L_ value for each dose of methacholine.

### Measurement of Lung Cytokines and Chemokines

Th1 (IL-2, IFN-γ)/Th2 (IL-4, IL-5) cytokines (including TNFα) and IL-13 were measured in the BALF by flow cytometry using CBA Flex Set kits (BD Biosciences, San Jose, CA) with a FACScan flow cytometer equipped with CellQuest Pro^TM^ (BD Biosciences) for data acquisition and FlowJo Software (Tree Star, Inc., Ashland, OR) for analysis or a FACSCelesta flow cytometer (for IL-13) with FACSDiva^TM^ Software (BD Biosciences) for data acquisition and analysis as described previously (Ge et al., 2013a). Eotaxin-1 (CCL11) and eotaxin-2 (CCL24) in the BALF were measured using ELISA kits (R & D Systems, Minneapolis, MN) according to the manufacturer’s recommendations. Results were expressed as pg/ml BALF in each case.

### Measurement of Total IgE

Total IgE in serum was measured using a mouse IgE ELISA MAX kit (BioLegend, San Diego, CA) as per the manufacturer’s recommendation.

### Analysis of Lung Eicosanoids

Eicosanoids in lung tissue were analyzed as previously reported (Al-Husseini et al., 2015) with minor modifications. Briefly, lung tissues were homogenized in ice-cold water in the presence of 50 µM indomethacin (Thermo Fisher Scientific) to obtain a 10% (w/v) solution. Volume of the tissue homogenate was brought up to 9 ml with ice-cold water followed by addition of 1 ml of 100% methanol to obtain a 10% (v/v) methanol in water solution. 100 µl of a stock of internal standards (Cayman Chemicals, Ann Arbor, MI, containing the following deuterated standards, (*d*_4_) thromboxane B2 (TxB2), (*d*_5_) 5(S),6(R)-lipoxin A4, (*d*_4_) LTB4, (*d*_8_) 5(S)-hydroxyeicosatetranoic acid (HETE), (*d*_8_) 15(S)-HETE, (*d*_6_) 20-HETE, (*d*_8_) 12(S)-HETE, (*d*_9_) PGD2, (*d*_9_) PGE2, (*d*_4_) 6-keto PGF1α (the stable metabolite of PGI2), (*d*_4_) 15-deoxy-Δ12,14-PGJ2, (*d*_5_) eicosapentaenoic acid (EPA), (*d*_5_) docosahexaenoic acid (DHA), (*d*_8_) AA, (*d*_5_) Resolvin D1, and (*d*_5_) Resolvin D2) and 50 µl of acetic acid were added to the samples and mixed by vortexing. The resultant mixture was centrifuged for 10 min at 4°C and 5,000 × *g*. Solid phase extraction was performed on the clarified supernatant using StrataX-C18 (Phenomenex, Torrance CA) solid phase extraction cartridges (30 mg). The analytes bound to the column were eluted in isopropanol *via* vacuum assisted percolation. The resultant eluant was vacuum dried, resuspended in 100 µl of 1:1 water:ethanol and analyzed *via* liquid chromatography tandem mass spectrometry. Chromatographic separation of eicosanoids was performed *via* a Shimadzu Nexera Ultra-high Performance Liquid Chromatography system (Shimadzu, Columbia, MD) using a Phenomenex Kinetex C18 2.1 × 100 mm reverse phase column (Phenomenex, Torrance CA). The eluting eicosanoids were analyzed *via* a Sciex QTRAP 6500+ Hybrid Triple Quadrupole Linear Ion Trap Mass Spectrometer (Sciex, Redwood City CA). Only eicosanoids demonstrating a signal to noise (s/n) ratio of at least 3 were considered for further analysis. Semi-quantitative analysis of the eicosanoids was undertaken *via* the method of isotope dilution. A full quantitation was impractical due to the commercial lack of analytical grade standards. For those eicosanoids where an internal standard was unavailable, the standard that is closest in structure was used for quantitation. Values are reported as ng of relevant eicosanoid per mg of tissue or as fold change relative to the level of the eicosanoid in control non-allergen challenged mice.

### Culture of BM-Derived Murine Eosinophils

Eosinophils were cultured from BM of naïve mice as described previously (Dyer et al., 2008; Bahaie et al., 2011). Cells between days 12–14 of culture that were 99% Hema 3-positive and expressed both MBP and Siglec-F determined as described (Bastan et al., 2018) were used in studies.

To examine the effect of inflammatory mediators on expression of COX-2, cells (∼5 × 10^6^/well) were cultured in medium containing 10% FBS and IL-4, TNFα or eotaxin-1 (all at 50 ng/ml, PeproTech, Rocky Hill, NJ) for 24 h at 37°C. Treated cells were lysed and used to evaluate COX-2 expression by q-PCR.

### q-PCR

Total RNA was extracted from eosinophils using *Quick*-RNA mini-prep (Zymo Research, Irvine, CA) and reverse-transcribed into cDNA using M-MLV Reverse Transcriptase (Promega, Madison, WI). Gene expression was examined by qPCR with primers specific for mouse COX-2 (Cheng et al., 2016) (FP: CAGGTCATTGGTGGAGAGGTGTAT; RP: CCAGGCACCAGACCAAAGACTT) and β-actin (Ha et al., 2013). Primers for both genes were synthesized by Integrated DNA Technologies (Coralville, IA). qPCR was performed using a Mx3000P qPCR System (Agilent, Santa Clara, CA). The amount of target mRNA was calculated based on its threshold cycle (Ct) normalized to the Ct of the housekeeping gene β-actin and results were expressed as fold change relative to expression of the target gene in untreated control cells using the ddCt method (2^−ΔΔCT^) (Schmittgen and Livak, 2008).

### Migration Assay

To examine the effect of PTUPB, celecoxib and *t*-TUCB on eosinophil migration, cells treated with each drug (5 or 10 µM, 20 min at 37ºC) or DMSO were added to the upper wells of Transwell^®^ 96-well plates (Corning Life Sciences, Tewksbury, MA) and migration towards murine eotaxin-1 (100 nM, PeproTech) in the lower wells of the chambers was assessed after 3–4 h at 37ºC (Ge et al., 2013b). The number of migrating cells was determined using an Olympus CK2 inverted microscope (×200 magnification). Cells in ten randomly selected non-overlapping fields were counted for each well and expressed as percent migration relative to vehicle-treated cells.

### Statistical Analysis

All experiments were repeated at least three times. Statistical significance between various groups was assessed by Brown-Forsythe and Welch ANOVA or ordinary one-way ANOVA (for analysis of lipid mediator levels to compare vehicle-treated allergen-challenged group with every other group and for analysis of COX-2 induction in inflammatory mediator-treated eosinophils compared to untreated control group) followed by Dunnett’s T3 *post hoc* test for multiple comparisons using GraphPad Prism Software (8.1.2). A *p* value < 0.05 was considered significant.

## Results

### Effect of PTUPB, Celecoxib and *t*-TUCB on Allergen-Induced Airway Cellular Inflammation and Eosinophilia

**Figure 1:**
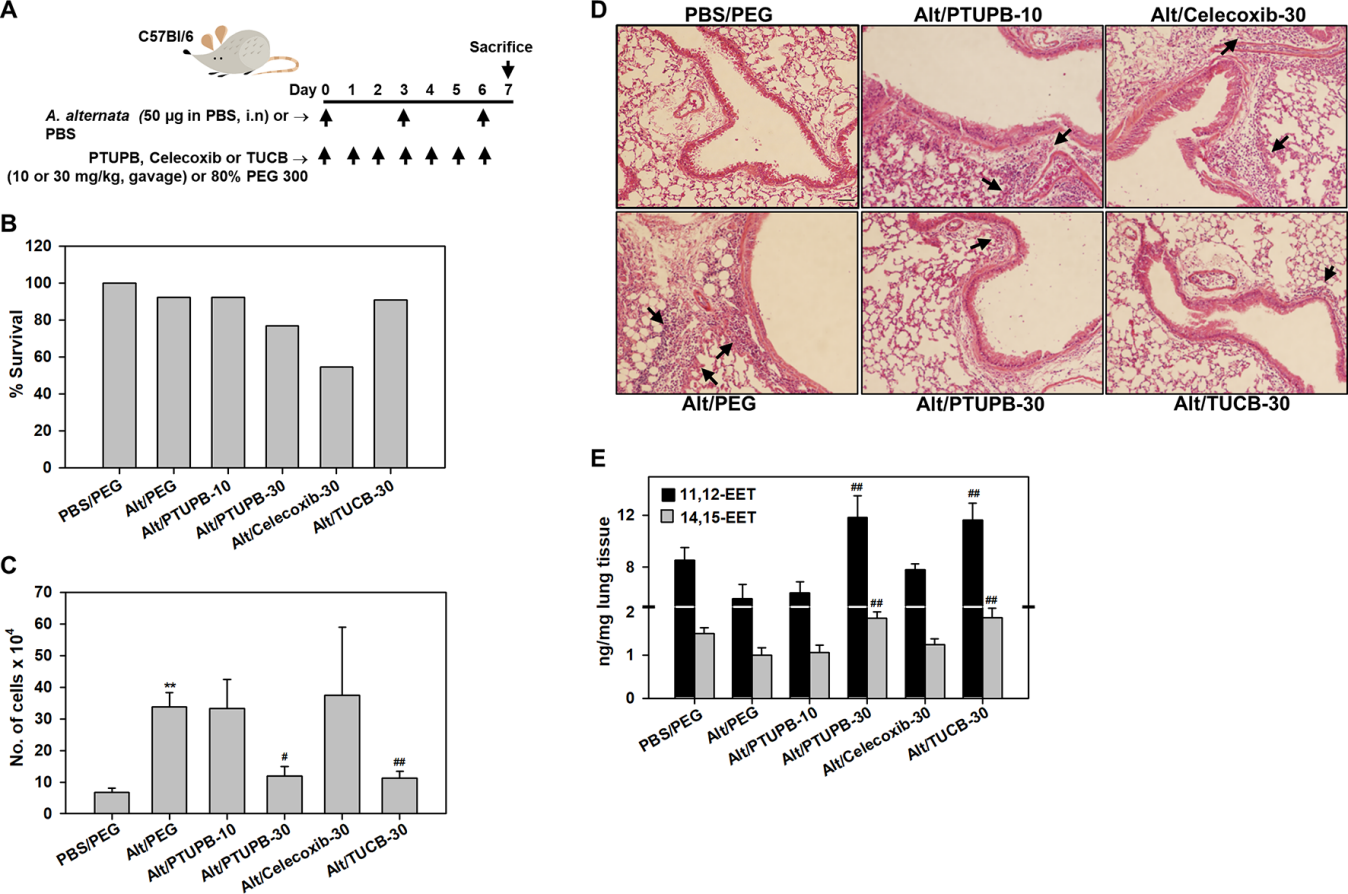
Effect of PTUPB, celecoxib and *t*-TUCB on allergen-induced airway cellular inflammation. **(A)** Outline of protocol for challenge with *A. alternata* and administration of PTUPB (dual sEH and COX-2 inhibitor), celecoxib (COX-2 inhibitor), *t*-TUCB (sEH inhibitor) or vehicle. i.n, intranasal. **(B)** Effect of inhibitors on survival of allergen-exposed mice. Alt, *A. alternata*. **(C)** Total cell counts in the BALF of control and *A. alternata*-challenged mice with or without administration of inhibitors. **(D)** H & E staining of lung tissue from mice identified in C. Representative image for each group is shown. Arrows indicate infiltrating inflammatory cells. **(E)** Concentration of EETs in lungs of control and *A. alternata*-challenged mice with or without administration of inhibitors. Scale bar, 50 µm in D. Data shown in C and E represent mean ± SEM. n = 6 mice for Alt/Celecoxib-30 group and 9-10 mice for all other groups in C and 5-6 mice/group in E. **p < 0.01 compared to PBS/PEG, ^#^p < 0.05 and ^##^ < 0.01 compared to Alt/PEG.

WT mice were challenged with *A. alternata*, an airborne fungal allergen, as outlined in **Figure 1A**, and examined for expression of COX-2 by immunohistochemical staining. While COX-2 expression in control (PBS-exposed) mice was noted mostly in the airway epithelium, exposure to *A. alternata* led to increased expression of COX-2 in the peribronchial areas of the lungs and was mostly associated with alveolar macrophages (**Supplementary Figure 1A**). Allergen-challenged mice were administered with PTUPB (sEH/COX-2 dual inhibitor), *t*-TUCB (sEH inhibitor) or celecoxib (COX-2 inhibitor) as shown in **Figure 1A**. A dosage of 30 mg/kg/day was selected for PTUPB based on previous studies in mice demonstrating its effectiveness at this dose without exerting any adverse effects (Zhang et al., 2014). Since this was a comparative study evaluating the effect of the three inhibitors, *t*-TUCB and celecoxib were also used at 30 mg/kg/day to maintain a consistent dosage. PTUPB was additionally tested at a lower dose of 10 mg/kg/day to determine the outcome of dual inhibition of the sEH and COX-2 pathways relative to inhibition of the two pathways independently (i.e., with TUCB or celecoxib) at 30 mg/kg/day. Administration of PTUPB at 10 mg/kg (PTUPB-10) or *t*-TUCB at 30 mg/kg (TUCB-30) had no effect on mortality in comparison to allergen-challenged mice that received vehicle alone. However, administration of celecoxib at 30 mg/kg (Celecoxib-30) or PTUPB at 30 mg/kg (PTUPB-30) resulted in 45% (5/11) and 23% (3/13) of the animals dying, respectively, before the end of the study (**Figure 1B**).

*A. alternata*-exposed mice treated with vehicle alone demonstrated a marked increase in recruitment of inflammatory cells to the airways relative to control mice (PBS-exposed and treated with vehicle) as indicated by the total number of cells in the BALF (**Figure 1C**) and cellular infiltration in lung tissue sections by H&E staining (**Figure 1D**). PTUPB-10 did not alter airway cellular inflammation in the allergen-challenged mice; a significant reduction in the number of BALF inflammatory cells was noted only at the higher dose, i.e., PTUPB-30, relative to mice that received vehicle alone (**Figure 1C**). This reduction in cellular inflammation was also evident in the lung tissue of these mice (**Figure 1D**). Celecoxib-30 did not affect airway cellular inflammation in the surviving mice. Administration of TUCB-30, on the other hand, significantly attenuated recruitment of inflammatory cells to BALF and lung tissue of allergen-challenged mice. Evaluation of lipid mediators in the lung tissue of these mice indicated that *A. alternata* challenge reduced levels of the anti-inflammatory lipid mediators 11,12- and 14,15-EET in the lungs compared to control mice, although the reduction was not statistically significant (**Figure 1E**). However, administration of PTUPB-30 and TUCB-30, but not PTUPB-10 or celecoxib-30, to allergen-challenged mice significantly increased levels of both these lipid mediators relative to allergen-challenged mice that received vehicle alone confirming the dose-dependent and specific inhibitory effect of these compounds on sEH. Level of 5,6-EET and 8,9-EET were extremely low and unaffected by *A. alternata* challenge with no further change after treatment with the inhibitors (data not shown).

**Figure 2:**
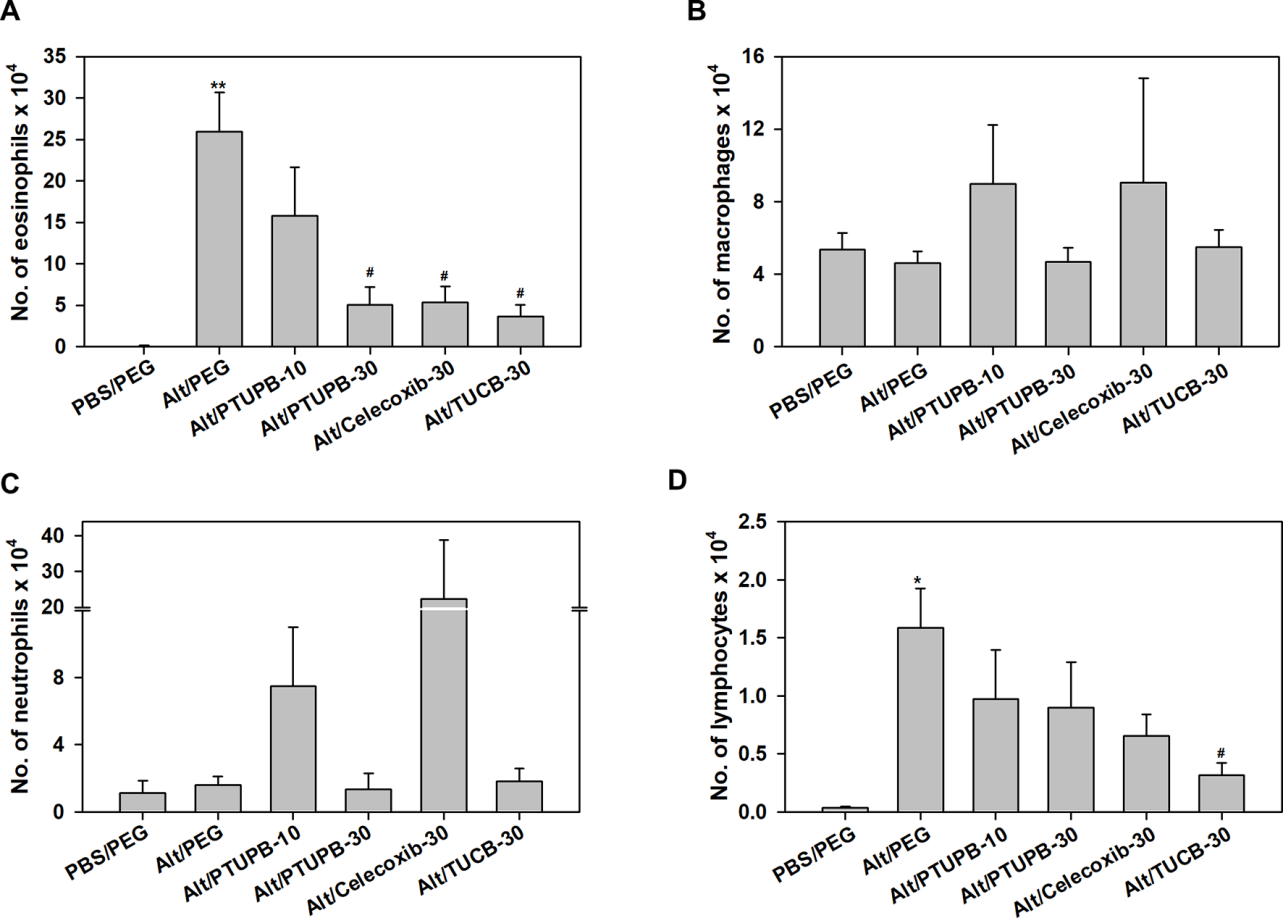
Airway eosinophil recruitment in allergen-challenged mice administered with PTUPB, celecoxib or *t*-TUCB. **(A)** Eosinophil, **(B)** macrophage, **(C)** neutrophil and **(D)** lymphocyte counts in the BALF of control and *A. alternata*-challenged mice with or without administration of PTUPB, celecoxib or *t*-TUCB. Data represent mean ± SEM. n = 6 mice for Alt/Celecoxib-30 group and 9-10 mice for all other groups. *p < 0.05 and ** < 0.01 compared to PBS/PEG, ^#^p < 0.05 compared to Alt/PEG.

Analysis of differential cell counts in the BALF demonstrated a marked reduction in recruitment of eosinophils in allergen-challenged mice treated with PTUPB-30, celecoxib-30, or TUCB-30 compared to vehicle-treated mice (**Figure 2A**). Exposure to *A. alternata* did not induce an influx of monocyte/macrophages or neutrophils to the airways, which remained unaffected by administration of PTUPB-30 or TUCB-30 (**Figures 2B, C**). The effect of PTUPB-10 and celecoxib-30 on the number of monocyte/macrophages and neutrophils in the BALF was variable, with some mice exhibiting increased numbers of these cell types relative to allergen-challenged mice treated with vehicle. This variable effect on the number of macrophages and neutrophils noted with PTUPB-10 and celecoxib-30 may explain the absence of an effect on total airway cellular inflammation by these compounds (**Figures 1C, D**). The number of lymphocytes in mice administered with PTUPB-10, PTUPB-30 and celecoxib-30 was lower than in vehicle-treated mice, although not statistically significant (**Figure 2D**). A significant reduction in the number of lymphocytes was noted only in mice that received TUCB-30.

**Figure 3:**
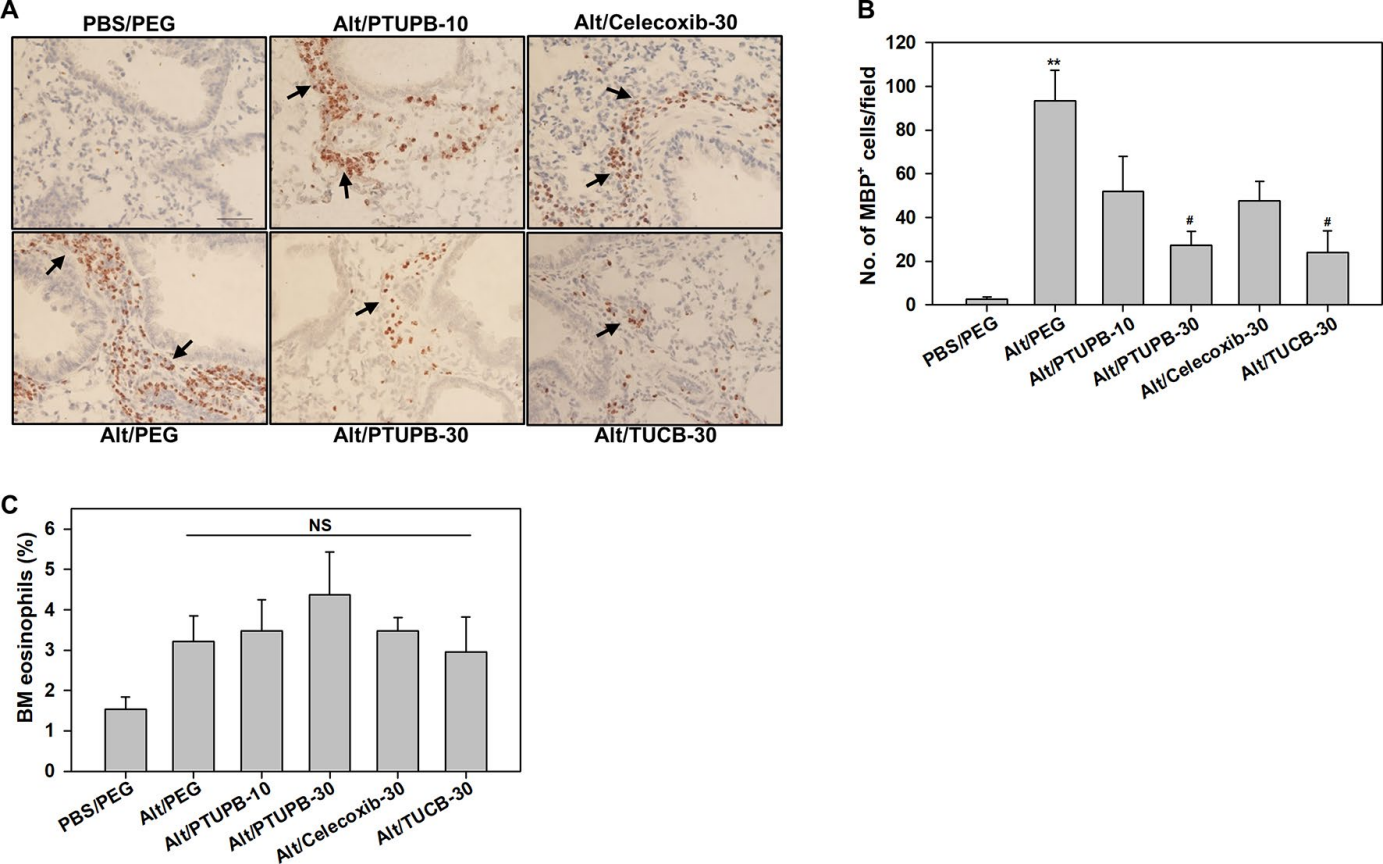
PTUPB and *t*-TUCB inhibit eosinophil recruitment in the lung tissue of allergen-challenged mice. **(A)** Immunohistochemical staining for expression of eosinophil-specific MBP (stained dark brown, black arrows) in the lung tissue of control and *A. alternata*-challenged mice with or without administration of PTUPB, celecoxib or *t*-TUCB. A representative image for each group is shown. Scale bar, 50 µm. **(B)** Quantitation of infiltrated lung tissue eosinophils (i.e., MBP-positive cells) in mice described in A. **(C)** Percentage of eosinophils in the BM of mice identified in A based on cell morphology after Hema3 staining. Data represent mean ± SEM. n = 6-7 mice/group in B, 6 mice for Alt/Celecoxib-30 group and 7-9 mice for all other groups in C. **p <0.01 compared to PBS/PEG, ^#^p < 0.05 compared to Alt/PEG. NS, not significant.

The effect of these inhibitors on lung tissue eosinophils was examined by immunohistochemistry based on expression of eosinophil-specific MBP. As expected, quantitation of MBP-positive cells demonstrated a significantly increased number of eosinophils in lung sections of vehicle-treated allergen-exposed mice relative to the number detected in control lung sections (**Figures 3A, B**). Consistent with the decreased number of eosinophils in the BALF (**Figure 2A**), there was a marked reduction in the number of MBP-positive cells detected in the lungs of allergen-challenged mice administered with PTUPB-30 or TUCB-30. A modest reduction in number of MBP-positive cells was also noted in mice that received PTUPB-10 or celecoxib-30, although the decrease was not statistically significant compared to vehicle-treated allergen-challenged mice in either case. The effect of these inhibitors on eosinophil proliferation in the BM was evaluated. Allergen-challenged mice had an increased number of eosinophils compared to unchallenged mice, albeit not statistically significant. None of the inhibitors significantly altered BM eosinophil proliferation relative to untreated mice (**Figure 3C**) suggesting that the effect of the inhibitors on reducing eosinophilia is at the level of cell recruitment. Overall, while allergen-challenged mice administered with PTUPB-30, celecoxib-30 or TUCB-30 demonstrated reduced eosinophil recruitment to a similar extent in the BALF, only PTUPB-30 and TUCB-30 treatment inhibited eosinophil infiltration in the lung tissue.

### Effect of PTUPB, Celecoxib, and *t*-TUCB on Th2 Cytokines, Eotaxins, and IgE

**Figure 4:**
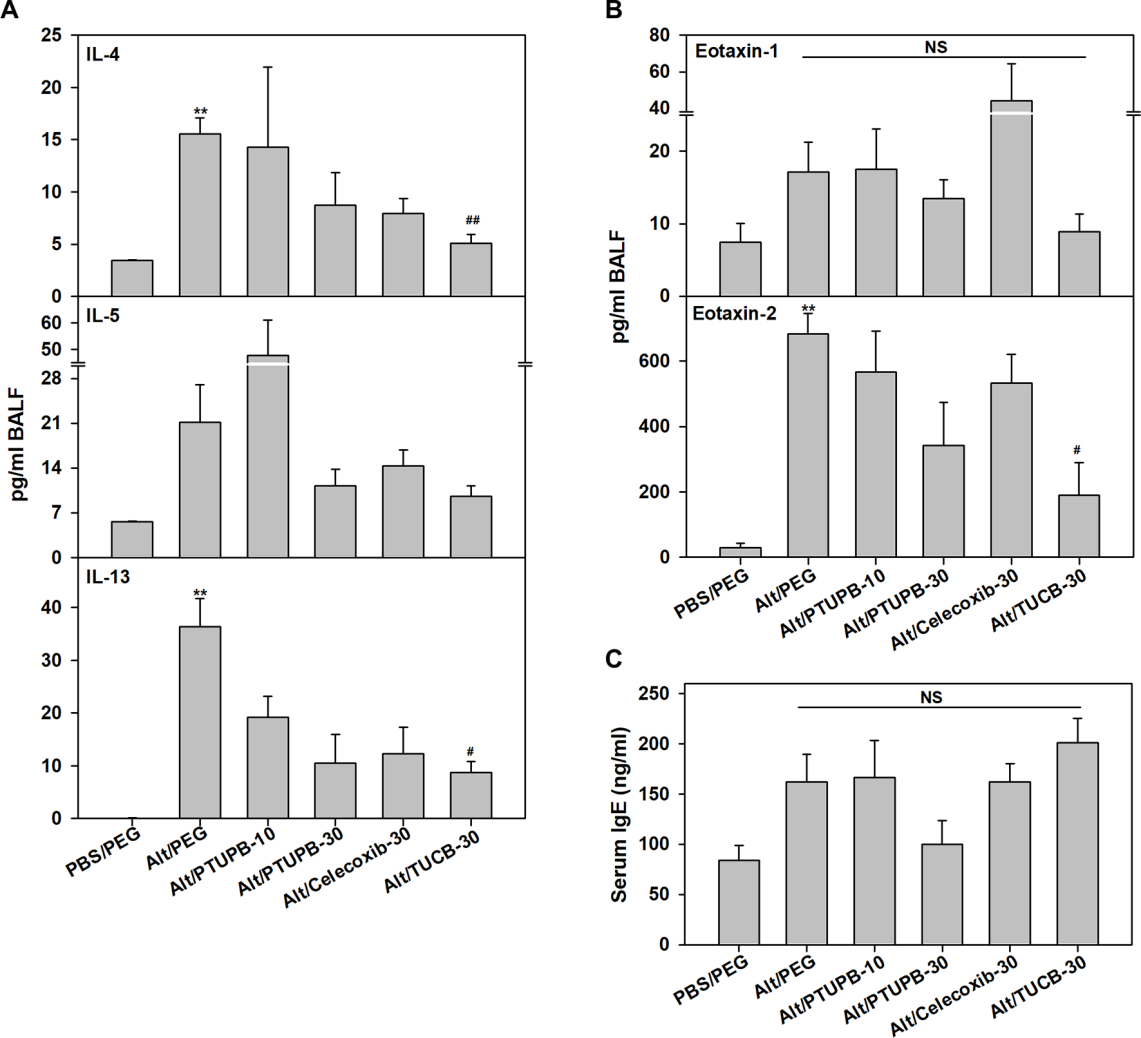
Effect of PTUPB, celecoxib and *t*-TUCB on Th2 cytokines, eotaxins and IgE. **(A)** Th2 cytokine levels in BALF from control and *A. alternata*-challenged mice with or without administration of PTUPB, celecoxib or *t*-TUCB. **(B)** Eotaxin-1 and -2 levels in BALF, and **(C)** total IgE levels in the serum of mice identified in A. Data represent mean ± SEM. n = 5-7 mice per group. **p <0.01 compared to PBS/PEG, ^#^p < 0.05 and ^##^p < 0.01 compared to Alt/PEG. NS, not significant.

AAI and asthma is associated with elevated Th2 cytokine levels (Lambrecht et al., 2019). IL-4, IL-5 and IL-13 levels in the BALF were evaluated. Compared to control PBS-exposed mice, allergen-challenged mice treated with vehicle alone had significantly higher levels of IL-4 and IL-13 with a modest increase in IL-5 (**Figure 4A**). Administration of PTUPB-10 did not alter IL-4 or IL-5 levels but moderately reduced IL-13 (not statistically significant). While PTUPB-30 and celecoxib-30 reduced IL-4, IL-5 and IL-13 levels compared to vehicle-treated allergen-challenged mice, the reduction was not statistically significant. On the other hand, in mice administered with TUCB-30, IL-4 and IL-13 levels were significantly lower compared to vehicle-treated mice. IL-5 levels were also lower in these mice, although the reduction was not significant. *A. alternata* challenge did not increase TNF-α levels compared to control mice and remained unaltered by administration of inhibitors, while IL-2 and IFN-γ levels were below detection limit in all groups (data not shown).

Since PTUPB-30, celecoxib-30, and TUCB-30 inhibited recruitment of eosinophils in the BALF, levels of the eosinophil-active chemokines, eotaxin-1 and -2 (Pope et al., 2005), were measured. Eotaxin-1 in allergen-challenged mice that received vehicle alone was marginally higher than in control mice and was not significantly altered by any of the inhibitors (**Figure 4B**). Eotaxin-2 levels were significantly higher in vehicle-treated allergen-challenged mice relative to control mice. PTUPB-10 and celecoxib-30 had no effect, while a marginal reduction in eotaxin-2 was noted with PTUPB-30 relative to vehicle-treated mice. TUCB-30 on the other hand, significantly inhibited eotaxin-2 levels (**Figure 4B**). Since elevated IgE is a distinguishing feature of allergic inflammation, we examined the effect of these inhibitors on allergen-induced IgE levels in the serum. Consistent with the allergic disease phenotype, IgE levels in allergen-challenged mice were higher than in control mice, although the increase was not statistically significant. None of the inhibitors significantly altered IgE levels in the allergen-challenged mice (**Figure 4C**).

### Effect of PTUPB, Celecoxib, and *t*-TUCB on Allergen-Induced Changes in Lung Lipid Mediators

**Figure 5:**
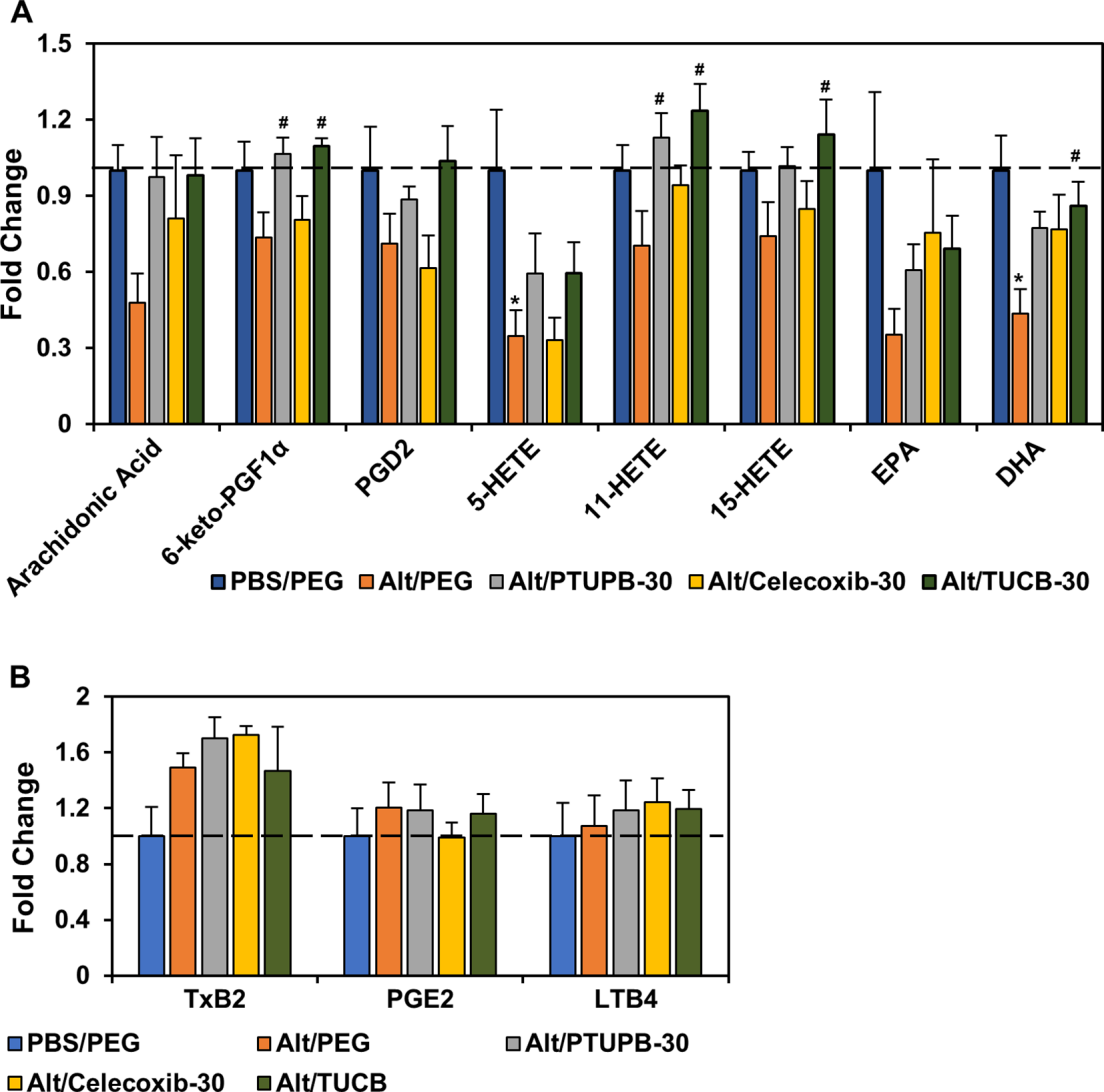
Effect of PTUPB, celecoxib and *t*-TUCB on allergen-induced changes in lung lipid mediators. Eicosanoid levels in control and *A. alternata*-challenged mice with or without administration of PTUPB, celecoxib or *t*-TUCB shown as fold change relative to unchallenged mice. Eicosanoids that were decreased by allergen exposure are shown in panel **(A)** and those that increased or remained unchanged are shown in panel **(B)**. n = 5-6 mice per group. *p < 0.05 compared to PBS/PEG, ^#^p < 0.05 compared to Alt/PEG.

Eicosanoids resulting from AA metabolism contribute to the pathogenesis of allergic asthma by playing both pro-inflammatory as well as anti-inflammatory roles (Sanak, 2016; Peebles, 2018). Eicosanoid levels in the lung tissue of allergen-challenged mice administered with PTUPB-30, celecoxib-30, *t*-TUCB-30 or vehicle alone were analyzed. Exposure to *A. alternata* reduced levels of AA as well as various COX and LOX metabolites such as 6-keto-PGF1α, PGD2, 11- and 15-HETE, and the omega 3 polyunsaturated fatty acid EPA, with a significant reduction noted in the levels of 5-HETE and DHA (**Figure 5A**). Administration of PTUPB-30 and TUCB-30 increased levels of all these lipid mediators, in some cases to levels noted in lungs of control mice. Level of 6-keto-PGF1α and 11-HETE were significantly higher in allergen-challenged mice treated with PTUPB-30 relative to allergen-challenged mice treated with vehicle alone. In the case of TUCB-30, levels of 6-keto-PGF1α, 11- and 15-HETE as well as DHA were significantly higher than in mice treated with vehicle alone. Administration of celecoxib-30 only increased levels of AA, 11-HETE, EPA, and DHA, although the increase was not found to statistically significant in the case of any of the mediators. *A. alternata* challenge increased TxB2 levels but not that of PGE2 or LTB4, which remained unaltered by administration of PTUPB-30, celecoxib-30 or TUCB-30 (**Figure 5B**).

### Effect of PTUPB, Celecoxib, and *t*-TUCB on Allergen-Induced AHR and Airway Structural Changes

**Figure 6:**
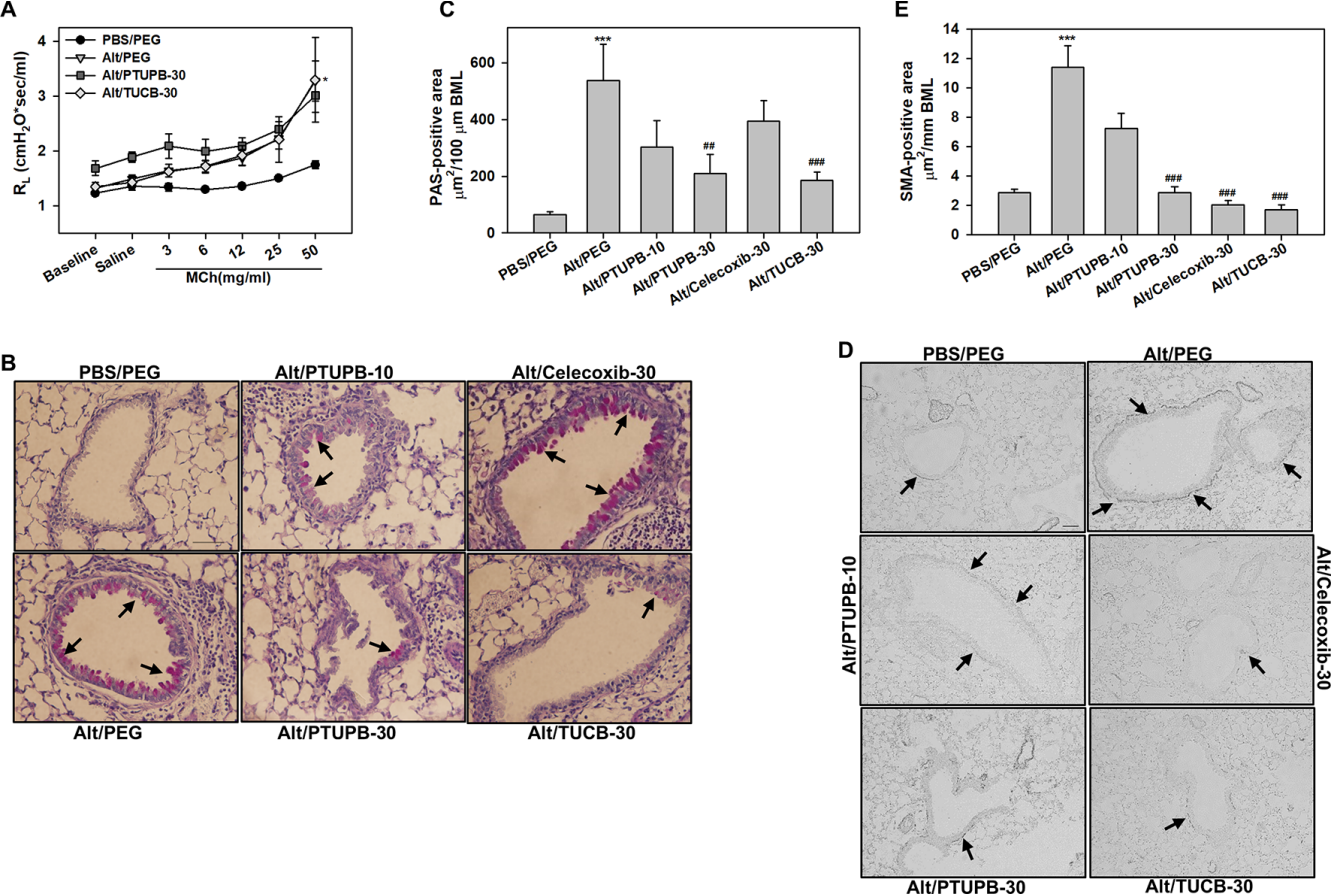
Administration of PTUPB or *t*-TUCB attenuates allergen-induced mucus secretion and smooth muscle thickening but not AHR. **(A)** Measurement of pulmonary resistance (*RL*) following exposure to increasing concentrations of aerosolized methacholine (MCh) in mechanically ventilated control and *A. alternata*-challenged mice with or without administration of PTUPB or *t*-TUCB. **(B** and **C)** Airway mucus secretion based on PAS staining (stained dark-pink, black arrows) in control and *A. alternata*-challenged mice administered with vehicle or with PTUPB, celecoxib or *t*-TUCB and quantitation of PAS-positive area in the airways, respectively. **(D** and **E)** Airway smooth muscle thickening in mice described in B assessed by immunohistochemical staining for α-SMA expression and quantitation of α-SMA-positive area, respectively. Scale bar, 50 µm. Data represent mean ± SEM. n = 5-7 mice per group. ***p < 0.001 compared to PBS/PEG, ^##^p < 0.01 and ^###^ < 0.001 compared to Alt/PEG.

AHR is one of the hallmark features of allergic asthma and is associated with structural changes in the airways such as increased mucus production and airway smooth muscle hyperplasia/hypertrophy that contribute to airflow obstruction (Bai and Knight, 2005). Vehicle-treated allergen-challenged mice displayed elevated AHR with significantly higher pulmonary resistance (R_L_) relative to control mice (**Figure 6A**). Administration of PTUPB-30 and TUCB-30 had no effect on allergen-induced AHR. The effect of celecoxib-30 could not be examined in the current study due to mice dying during the procedure thus resulting in small sample size. Associated with the elevated AHR, vehicle-treated allergen-challenged mice exhibited significantly increased airway mucus secretion and smooth muscle thickening compared to control mice based on PAS staining and α-SMA immunohistochemical staining, respectively (**Figures 6B–E**). Despite the lack of an effect on AHR, administration of PTUPB-30 or TUCB-30 strongly inhibited allergen-induced airway mucus production (**Figures 6B, C**) and smooth muscle hyperplasia (**Figures 6D, E**) in these mice. Interestingly, while substantial levels of mucus secretion were detectable in the airways of allergen-challenged mice that received celecoxib-30, smooth muscle thickening was significantly inhibited.

### Effect of PTUPB, Celecoxib, and *t*-TUCB on Eosinophil Migration

**Figure 7:**
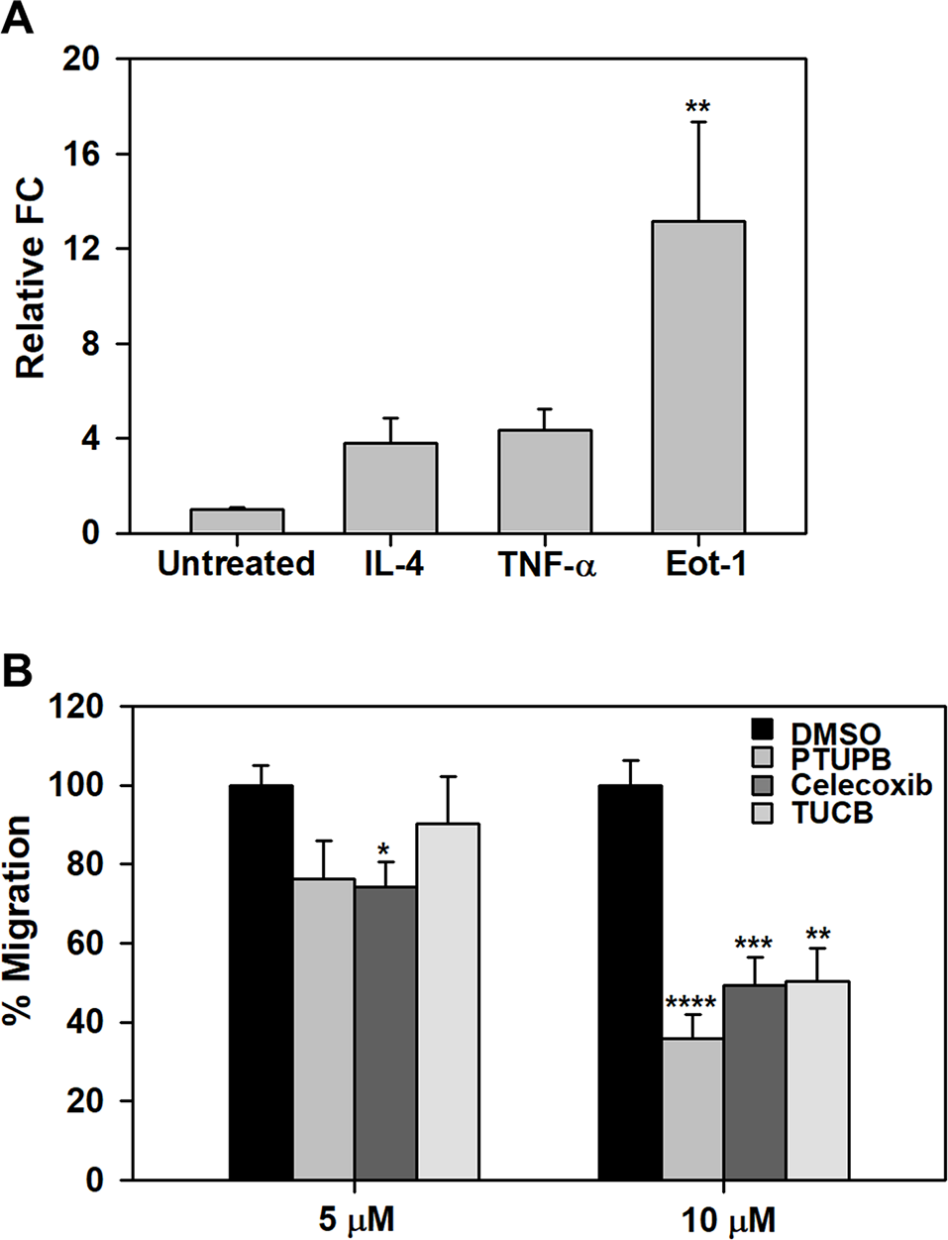
PTUPB, celecoxib and *t*-TUCB inhibit eosinophil migration. **(A)** Effect of pro-inflammatory mediators on expression of COX-2 in mouse eosinophils by qPCR. **(B)** Migration of eosinophils treated with PTUPB, celecoxib or *t*-TUCB at the indicated doses or vehicle alone towards eotaxin-1 in Transwell^®^ plates. Combined data (mean ± SEM) of three experiments in duplicate is shown. *p < 0.05, **p < 0.01, ***p < 0.001 and ****p < 0.0001 relative to untreated cells in A and DMSO in B.

We have previously demonstrated that pro-inflammatory mediators such as TNF-α and eotaxin-1 induce expression of sEH in eosinophils (Bastan et al., 2018). Here, we examined the effect of IL-4, TNF-α or eotaxin-1 on expression of COX-2 in BM eosinophils by qPCR. IL-4 and TNF-α marginally increased COX-2 expression in eosinophils. Eotaxin-1 on the other hand significantly induced COX-2 expression in these cells (**Figure 7A**). Next, we examined whether PTUPB, celecoxib or *t*-TUCB exert a direct effect on eosinophils migration. Celecoxib significantly inhibited eotaxin-1-induced eosinophil migration at 5 and 10 µM, while a significant inhibition of migration was noted at a concentration of 10 µM with PTUPB and *t*-TUCB (**Figure 7B**).

## Discussion

COX-2 expression in asthmatic subjects (Sousa et al., 1997; Profita et al., 2003) and in models of AAI (Oguma et al., 2002; Shiraishi et al., 2008; Herrerias et al., 2009), including the current study (**Supplementary Figure 1A**), is increased relative to controls. Although it is recognized that products of the COX pathway, e.g., PGD2, play a pro-inflammatory role in AAI (Shiraishi et al., 2008; Domingo et al., 2018), selective inhibition of COX-2 has yielded mixed outcomes towards resolution of airway inflammation (reviewed in Peebles, 2018). Like COX-2, sEH expression is increased in asthmatic airways (Morin et al., 2010) and lungs of allergen-challenged mice (**Supplemental Figure 1B**). Inhibition of sEH with *t*-TUCB attenuates various aspects of AAI (Yang et al., 2015) and food allergen-driven GI inflammation (Bastan et al., 2018), specifically allergen-induced eosinophilia, thus demonstrating its pro-inflammatory role in allergic inflammation. Eosinophilia is a hallmark of airway inflammation in asthma (George and Brightling, 2016). Eosinophils are a source of various cytokines, chemokines, growth factors and highly cytotoxic cationic granule proteins that are secreted upon activation (Davoine and Lacy, 2014) and known to exert various pro-inflammatory effects (reviewed in Acharya and Ackerman, 2014). Further, eosinophils are thought to contribute to asthma exacerbation (Nakagome and Nagata, 2018). Thus, identifying antagonists for specific mediators and/or pathways involved in promoting airway eosinophilic inflammation is critical for the identification of novel therapeutic strategies.

The efficacy of dual sEH/COX-2 inhibition with PTUPB relative to selective inhibition of COX-2 with celecoxib or sEH with *t*-TUCB independently in reducing eosinophilia and airway inflammation in a clinically relevant model of AAI induced by the fungal allergen *A. alternata* (Gabriel et al., 2016) was investigated. In our studies, increased mortality was noted in allergen-challenged mice administration with celecoxib (45%), a finding not reported previously. Only a slight increase in mortality was noted in mice administered with the dual sEH/COX-2 inhibitor PTUPB at the higher dose, while *t*-TUCB at the higher dose did not affect survival. While this effect of COX-2 inhibition on survival of mice is not entirely clear, previous studies have shown increased blood pressure and leukocyte adherence to the endothelium in animal models (Muscará et al., 2000) and increased cardiovascular disease in patients with suppression of COX-2, which may be due to inhibition of the synthesis of the vasodilator PGI2 (FitzGerald, 2004). Celecoxib has been administered to mice at doses close to or higher than 30 mg/kg/day with no report of toxicity, albeit in models other than AAI (Park et al., 2008; Zhang et al., 2017). Our observation of increased mortality in allergen-challenged mice treated with celecoxib raises the question whether inhibition of COX-2 in a setting of AAI/asthma might be associated with harmful effects. Additional studies are needed to understand the effect of selective inhibition of COX-2 on survival during AAI. BALF eosinophilia was significantly reduced in mice administered with PTUPB at the higher dose, celecoxib and *t*-TUCB, while in the lung tissue only PTUPB and *t*-TUCB, but not celecoxib, strongly inhibited eosinophil recruitment. It is possible that administration of celecoxib does not affect allergen-induced eosinophil recruitment to the lungs and the recruited eosinophils are retained in the lung tissue, thus accounting for the lower eosinophil numbers detected in the BALF of these mice. Other studies have reported that selective COX-2 inhibition with lumiracoxib (Swedin et al., 2010) or COX-2 knock-down (Carey et al., 2003) does not affect airway eosinophilia during AAI.

EETs derived from AA metabolism *via* the CYP450 enzyme pathway are well-known anti-inflammatory mediators (Morisseau and Hammock, 2013; Christmas, 2015). In the current study, levels of 11,12- and 14,15-EETs in the lung tissue were reduced by *A. alternata* challenge. Administration of PTUPB at the higher dose or *t*-TUCB, but not PTUPB at the lower dose or celecoxib, significantly increased levels of these EETs. Importantly, this increase in levels of 11,12- and 14,15-EETs was associated with decreased lung tissue eosinophilia. Additionally, Th2 cytokines IL-4, IL-5, and IL-13 were lower in the BALF of mice administered with PTUPB at the higher dose, celecoxib or *t*-TUCB, although a statistically significant reduction was noted only in IL-4 and IL-13 levels in allergen-challenged mice administered with *t*-TUCB. This may be linked to the observation that lymphocyte counts were significantly lower only in the TUCB-treated mice but not in PTUPB- or celecoxib-treated mice. Levels of the eosinophil-specific chemokines eotaxin-1 and -2 were also reduced in *t*-TUCB-treated mice, with a marked decrease noted for eotaxin-2. On the other hand, both eotaxins were moderately reduced with PTUPB at the higher dose but remained elevated in celecoxib-treated mice. It is likely that COX-2 inhibition does not affect expression of eotaxins, which may be another contributing factor to the higher number of eosinophils in the lung tissue of celecoxib-treated mice. Our findings with administration of *t*-TUCB are similar to those from a previous study where OVA-challenged mice administered with *t*-TUCB demonstrated increased levels of EETs (5,6-, 11,12-, and 14, 15-) associated with decreased eosinophilia and reduced IL-4 and IL-5 levels (Yang et al., 2015). Overall, our studies suggest that PTUPB at the higher dose and *t*-TUCB are equally effective in reducing eosinophilic inflammation, while *t*-TUCB alone but not PTUPB or celecoxib appears to be more effective at significantly reducing levels of specific Th2-promoting inflammatory mediators, i.e., IL-4, IL-13 and eotaxin-2.

Exposure to *A. alternata* resulted in reduction of various lipid mediators (AA, 6-keto-PGF1α, PGD2, 11,12-EET, 14,15-EET, 5-, 11- and 15-HETE, EPA and DHA) in lung tissue homogenates. Our findings with respect to levels of some of these lipid mediators, e.g., PGD2, 6-keto-PGF1α, are different (lower in allergen-challenged mice) than those observed in OVA- (Swedin et al., 2010) or HDM-induced (Herrerias et al., 2009) models of AAI. These differences may be due to the nature of the allergen (fungal versus non-fungal), duration of allergen challenge (6 days versus 10-46 days) and sample used for measurement of lipid mediators (lung tissue versus BALF). Nonetheless, administration of PTUPB at the higher dose or *t*-TUCB increased levels of all of these mediators, in some cases to levels noted in lungs of control mice and significantly higher than in allergen-challenged mice that received vehicle alone. Celecoxib on the other hand, increased levels of only some of the mediators (AA, 11-HETE, EPA, and DHA), although not in a statistically significant manner. In the context of eosinophil recruitment, we have previously shown that 11,12-EET, which was significantly increased after treatment with PTUPB at the higher dose or *t*-TUCB, can directly inhibit eotaxin-induced migration of eosinophils (Bastan et al., 2018). 14,15-EETs, which were also elevated after treatment with PTUPB at the higher dose or *t*-TUCB, have been shown to exert anti-inflammatory effects in human bronchi by inhibiting IκBα degradation resulting in lower NFκB-dependent transcription (Morin et al., 2008). In the current study, 6-keto-PGF1α, the stable metabolite of PGI2, was restored to levels seen in the lungs of control unchallenged mice by PTUPB at the higher dose and *t*-TUCB (but not celecoxib). Studies in experimental models suggest that PGI2 restrains AAI by inhibiting dendritic cell-mediated immune activation effects including inhibition of Th2 cytokines from Th2 polarized CD4^+^ cells (Debeuf and Lambrecht, 2018). Lipid mediators such as AA, HETEs, EPA and DHA can play an important role in regulating acute inflammation by serving as a source of potent lipid-derived pro-resolving mediators (Serhan, 2014; Antonio Gonçalves et al., 2018). While lipoxin A4, resolvins D1 and D2 were not detected at measurable levels in this model, it is possible that other pro-resolving mediators that were not analyzed in this study could be generated from these lipid mediators. Nonetheless, the reversal of various lipid mediator levels to that noted in non-allergen-challenged controls by PTUPB at the higher dose and *t*-TUCB appears to be directly associated with attenuation of airway cellular inflammation and suppression of a Th2 phenotype.

Regardless of their ability to reduce eosinophil recruitment to the airways significantly, PTUPB at the higher dose and *t*-TUCB failed to inhibit allergen-induced AHR. Our observation with *t*-TUCB is in contrast to previous studies in an OVA-induced model of AAI where administration of *t*-TUCB at 1 mg/kg rescued methacholine-induced AHR in allergen-challenged mice (Yang et al., 2015). This difference may be due to the type and duration of allergen exposure as well as concentration, frequency and route of administration of the inhibitor. Previous studies have shown that 14,15-EET can relax airway smooth muscle cells (Benoit et al., 2001) and that inhibition with a selective sEH inhibitor [12-(3-adamantan-1-yl-ureido)-dodecanoic acid] different than the one used in the current study significantly reduces TNFα-induced hyperreactivity of human bronchi to methacholine (Morin et al., 2010). Although levels of 14,15-EETs were increased in allergen-challenged mice after administration of PTUPB at the higher dose or *t*-TUCB in the current study, it is possible that the concentrations are not sufficient to exert a suppressive effect on AHR. Further TxB2, a metabolite of TxA2, was increased in allergen-challenged mice and remained elevated after administration of inhibitors. Studies in animal models of AAI and asthmatics using TxA2 antagonists suggest a role for this lipid mediator in allergen-induced airway reactivity (reviewed in Peebles, 2018). Additional studies are needed to further understand the persistence of AHR in spite of attenuated eosinophilic inflammation. Alternatively, airway inflammation as noted in the BALF/lung tissue and AHR may be dissociated, and differently regulated, events during *A. alternata*-induced AAI. Despite the sustained AHR in allergen-challenged mice treated with PTUPB or *t*-TUCB, airway mucus secretion and smooth muscle thickening, both distinguishing features associated with increased airway reactivity, were significantly inhibited. Interestingly, celecoxib had no effect on allergen-induced airway mucus secretion but reduced airway smooth muscle mass. These findings are somewhat different from those reported in an OVA-model of AAI using a different COX-2-selective inhibitor (Swedin et al., 2010), wherein mice treated with the inhibitor continued to show not only increased airway mucus secretion but also smooth muscle mass along with exaggerated AHR. Based on various studies in the literature, it is clear that a particular eicosanoid can have a pro-inflammatory or anti-inflammatory effect depending on the cell type. Further, *in vivo* a certain cell type is exposed to both pro- and anti-inflammatory eicosanoids and the cellular response is likely to be determined by a balance between the effects.

Inflammatory mediators such as IL-4, TNFα, and/or eotaxin-1 induced expression of COX-2 (in the present study) and sEH (as shown previously Bastan et al., 2018) by eosinophils. Induction of COX-2 expression by inflammatory mediators has been reported in other cells of the airways such as smooth muscle and epithelial cells and is associated with increased release of PGs from these cells (Belvisi et al., 1997). Increased expression of COX-2 and/or sEH by eosinophils as noted in the present study can lead to increased synthesis of eosinophil-activating mediators. For example, studies have shown that eosinophils synthesize and secrete PGD2, which in turn can act in an autocrine manner to activate eosinophils (Luna-Gomes et al., 2011). In the current study, we found that treatment of eosinophils with PTUPB, celecoxib or *t*-TUCB inhibits eotaxin-induced migration *in vitro*, with maximum inhibition noted with PTUPB. We have previously shown that sEH inhibition with *t*-TUCB inhibits eotaxin-induced migration of eosinophils *in vitro* (Bastan et al., 2018). *t*-TUCB has been shown to exert anti-inflammatory effects on human monocytes by increasing the EET to DiHET ratio (Sanders et al., 2012) and also blocking migration in response to MCP-1 (Kundu et al., 2013). Thus, blockade of eosinophil migration by PTUPB and *t*-TUCB at a cellular level may contribute to the overall reduction in eosinophilia noted in allergen-challenged mice administered with these inhibitors. The ability of celecoxib to inhibit eosinophil migration effectively *in vitro* but not *in vivo* is not entirely clear and may in part be due to the elevated eotaxin levels in allergen-challenged mice even after administration of this inhibitor.

In summary, dual inhibition of the COX-2 and sEH pathways was as effective as inhibition of sEH alone with respect to reducing eosinophilia, restoring various lipid mediator levels and decreasing airway structural changes but not with inhibiting levels of Th2-promoting inflammatory mediators in a significant manner (IL-4, IL-13, and eotaxin-2) in an *A. alternata*-induced model of AAI. Inhibiting the COX-2 pathway alone had only moderate or no effect on several of these features associated with *A. alternata*-induced AAI. While these studies provide valuable information on the role of sEH/COX-2 dual inhibition in preventing the development of eosinophilia and several features of AAI, future studies will focus on investigating the therapeutic effect of dual sEH/COX-2 inhibition in a model of established allergic airway disease. However, since allergen-induced AHR remained elevated, dual inhibition of the COX-2 and sEH pathways may be useful in the treatment of conditions where eosinophilic inflammation with no bronchial reactivity, e.g., eosinophilic esophagitis, dermatologic disorders, and pain-associated inflammation co-exist, with the additional advantage of reducing side effects associated with COX inhibitors.

## Data Availability Statement

The datasets generated for this study are available on request to the corresponding author.

## Ethics Statement

The animal study was reviewed and approved by Institutional Animal Care and Use Committee at the University of Minnesota.

## Author Contributions

MD, SR-S, and YG performed the experiments and analyzed the data. DW and NK performed the lipid mediator measurement using mass spectrometry and the data analysis. JY, SH, and BH contributed to study design pertaining to drug administration to animals and provided essential reagents (sEH and dual sEH/COX-2 inhibitors). PS assisted with study design, manuscript review and secured funding for the studies. SR designed experiments, prepared figures and wrote the manuscript. All authors read and approved the final manuscript.

## Funding

This work was supported by the University of Minnesota College of Veterinary Medicine (to PS) and in part by NIEHS (River Award R35ES030443 to BDH) and NIH (HD087198 to DW). The content is solely the responsibility of the authors and does not necessarily represent the official views of the NIH.

## Conflict of Interest

The authors declare that the research was conducted in the absence of any commercial or financial relationships that could be construed as a potential conflict of interest.

The reviewer, AH, declared a past co-authorship with one of the authors, BH, to the handling editor at the time of the review.
