# Supplementary figures and images for: Effect Of Dual sEH/COX-2 Inhibition on Allergen-Induced Airway Inflammation
Source: Front Pharmacol. 2019 Sep 27;10:1118. doi: 10.3389/fphar.2019.01118 (PMC6777353; doi:10.3389/fphar.2019.01118)

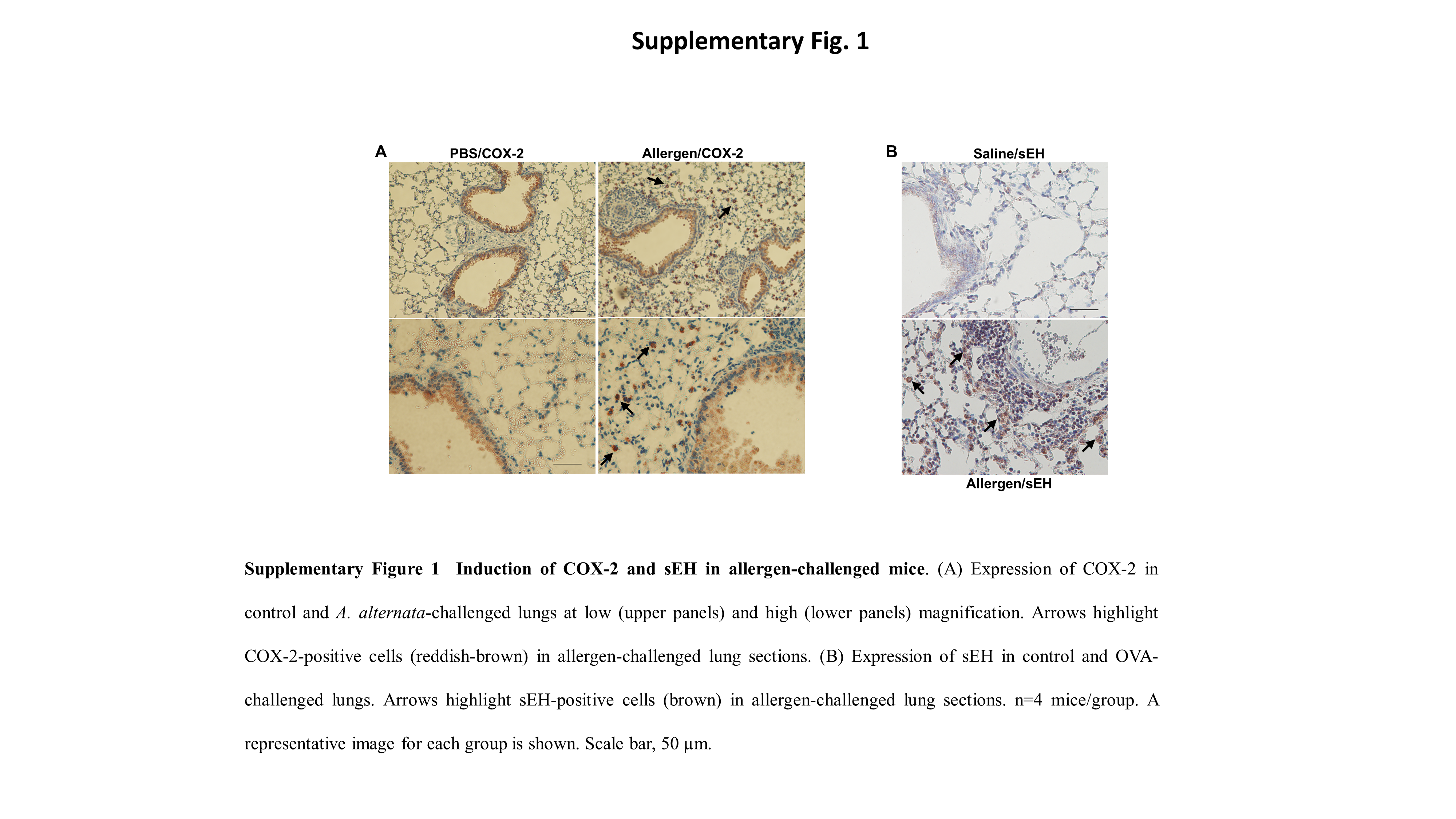

Supplement: Supplementary file 1 [file Image_1.tif]
